# How Substitution Combines with Non-Covalent Interactions to Modulate 1,4-Naphthoquinone and Its Derivatives Molecular Features—Multifactor Studies

**DOI:** 10.3390/ijms221910357

**Published:** 2021-09-26

**Authors:** Michał Pocheć, Karol Kułacz, Jarosław J. Panek, Aneta Jezierska

**Affiliations:** Faculty of Chemistry, University of Wrocław, ul. F. Joliot-Curie 14, 50-383 Wrocław, Poland; michal.pochec@chem.uni.wroc.pl (M.P.); karol.kulacz@chem.uni.wroc.pl (K.K.); jaroslaw.panek@chem.uni.wroc.pl (J.J.P.)

**Keywords:** 1,4-naphthoquinone derivatives, substituent effect, DFT, HOMA, Hirshfeld atomic charges, cSAR, CPMD

## Abstract

Substitution is well-known to modulate the physico-chemical properties of molecules. In this study, a combined, multifactor approach was employed to determine a plethora of substitution patterns using –Br and –O-H in 1,4-naphthoquinone and its derivatives. On the basis of classical Density Functional Theory (DFT), 25 models divided into three groups were developed. The first group contains 1,4-naphthoquinone and its derivatives substituted only by –Br. The second group consists of compounds substituted by –Br and one –O-H group. As a result of the substitution, an intramolecular hydrogen bond was formed. The third group also contains –Br as a substituent, but two –O-H groups were introduced and two intramolecular hydrogen bonds were established. The simulations were performed at the ωB97XD/6-311++G(2d,2p) level of theory. The presence of substituents influenced the electronic structure of the parent compound and its derivatives by inductive effects, but it also affected the geometry of the **2** and **3** groups, due to the intramolecular hydrogen bonding and the formation of a quasi-ring/rings. The static DFT models were applied to investigate the aromaticity changes in the fused rings based on the Harmonic Oscillator Model of Aromaticity (HOMA). The OH stretching was detected for the compounds from groups **2** and **3** and further used to find correlations with energetic parameters. The evolution of the electronic structure was analyzed using Hirshfeld atomic charges and the Substituent Active Region (cSAR) parameter. The proton reaction path was investigated to provide information on the modulation of hydrogen bridge properties by diverse substitution positions on the donor and acceptor sides. Subsequently, Car–Parrinello Molecular Dynamics (CPMD) was carried out in the double-bridged systems (group **3**) to assess the cooperative effects in double –O-H-substituted systems. It was determined that the –O-H influence on the core of the molecule is more significant than that of –Br, but the latter has a major impact on the bridge dynamics. The competitive or synergic effect of two –Br substituents was found to depend on the coupling between the intramolecular hydrogen bridges. Thus, the novel mechanism of a secondary (cooperative) substituent effect was established in the double-bridged systems via DFT and CPMD results comparison, consisting of a mediation of the bromine substitutions’ influence by the cooperative proton transfer events in the hydrogen bridges.

## 1. Introduction

The substituent effect (SE) is one of the major factors influencing various features of molecules [1,2,3,4,5,6,7,8,9,10,11]. Traditionally, SE was described quantitatively by Hammett constants (σ) [12,13]. However, it was noticed that when the reaction site significantly differs from COOH/COO${}^{-}$, the Hammett constants do not work properly; therefore, new σ constants were proposed [13]. Over the years, there have appeared in the literature modifications of the σ constants, e.g., the Taft equation [14]. New descriptors have been developed, based on quantum-chemistry models, e.g., Substituent Active Region (cSAR) [15], Substituent Effect Stabilization Energy (SESE) [16,17], pi-Electron-Donor-Acceptor/sigma-Electron-Donor-Acceptor (pEDA/sEDA) [18], Nucleus Independent Chemical Shift (NICS) [19], and Harmonic Oscillator Model of Aromaticity (HOMA) [20]. These and many other descriptors have been widely employed by contemporary science to describe quantitatively/qualitatively the SE effect—see [5] and references therein.

In the history of science, much spectacular evidence of SE can be found. One example could be considered in the current study, e.g., 1,4-naphthoquinone [21] substituted by two hydroxyl groups introduced a new compound—naphthazarin [22]—which can be obtained experimentally or from natural sources. 1,4-Naphthoquinone and its derivatives have been of significant interest to the scientific community in recent years due to their various molecular features [23,24,25]. Let us discuss naphthazarin (5,8-dihydroxy-1,4-naphthoquinone), gaining significance due to its chemical composition and molecular properties. Containing two rings, it is highly susceptible to diverse substitution patterns, resulting in new compounds with many interesting features. Its derivatives can be synthesized or acquired from natural sources [26,27,28], and they are important in numerous applications. Members of this group of compounds exhibit antifungal and antibacterial properties [29,30], can be used as very potent pesticides [31] and have many potential medical applications, spanning from anti-inflammatory pharmacotherapy [32] to oncology [33,34]. Due to all of the aforementioned features, there is still the necessity to characterize the naphthazarin derivatives, as well as great potential in predicting them via computational studies [35,36].

The presence of two hydroxyl groups in naphthazarin results in the formation of double intramolecular hydrogen bonds [22]. The nature of hydrogen bonds was investigated experimentally and theoretically, see, e.g., Refs. [37,38,39]. The presence of this kind of bonding introduces non-covalent interactions into the molecule. An intramolecular hydrogen bond influences the chemical composition of the molecule by, e.g., quasi-ring formation. It generally stabilizes the structure, evidenced by its spectroscopic properties [40,41]. The importance and role of hydrogen bonds are not in question; there is still, however, a need to understand their nature, especially taking into account the cases of unconventional hydrogen bonds [42,43]. As is well-known, they are ubiquitous and cannot be neglected in the discussion of factors decisive for the composition of bulk materials: both liquid and solid states. Additionally, they are important elements of many processes at the molecular level [44,45,46,47]. In summary, it is evident that hydrogen bonds (intra- or intermolecular) are non-covalent interactions of great significance in many branches of contemporary science [48,49,50,51,52].

It is necessary to mention that methods of modification have a significant role in the smart design of materials—they allow new systems with desired properties to be obtained. A good example of a substitution pattern’s influence on the system properties is provided by phenylene-diamide copolymers. Nomex (poly[phenylene-1,3-diamide]) and Kevlar (poly[phenylene-1,4-diamide]) are polyamides with high strength, stiffness, and low density [53], and as such are applied in many demanding industry areas. These materials are based on the substitutional isomeric forms of substrates, which causes differences in polymerization spatial patterns, resulting in differences in a product’s three-dimensional (3D) structure and different properties; Nomex/Conex is a meta-aramide with higher elongation and solubility in organic solvents, and Kevlar/Twaron is a para-aramide with a five-fold higher modulus and tensile strength [54,55]. A smart design demands prediction of those properties, and the need arises to deliver those predictions on the basis of computational chemistry, at least as preliminary results.

**Figure 1:**
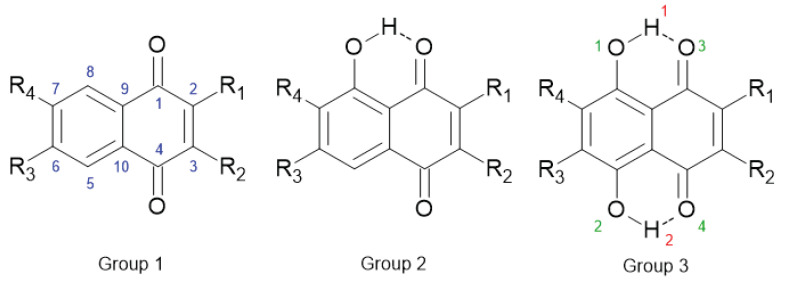
1,4-Naphthoquinone and its derivatives with atom numbering scheme: carbon—blue, oxygen—green and bridge hydrogen—red. The dotted lines indicate the presence of intramolecular hydrogen bonds. R${}_{1}$, R${}_{2}$, R${}_{3}$, R${}_{4}$ indicate the position of –H and –Br. The details of the substitution are presented in Table 1.

In this study, we will concentrate on a wide array of bromine- and hydroxyl-substituted compounds belonging to one of three groups of 1,4-naphthoquinone derivatives, presented in Figure 1 and Table 1. Groups **2** and **3** contain hydroxyl groups—the former in position 8 and the latter in positions 8 and 5 (naphthazarin belongs here, as the **3a** compound). This allows opportunities for not only SE, but also studies on non-covalent interaction impacts.

In this spirit, the choice of bromine derivatives is also associated with the presence of the hydrogen bridge. The –Br substituent has relatively mild Hammett constants ($\sigma_{p}$ = 0.23, $\sigma_{m}$ = 0.39), but its field and resonance parameters for gas-phase proton transfer equilibria have opposite signs ($\sigma_{F}$ = +0.45, $\sigma_{R^{+}}$ = −0.15, see Table IX of Ref. [1]). This is connected with the peculiarity of halogen atoms, which are electron-withdrawing, and thus deactivating, but by the virtue of their resonance back-donation effect, they are para- and ortho-directors in classical electrophilic substitution reactions. Such a mixed mode of action makes the –Br substituent interesting while modulating features of the bridged proton pathway. Therefore, we have looked for some correlations in the bridged proton dynamics and –Br substituent position in the core part of 1,4-naphthoquinone. Further, the relatively large size of the –Br substituent makes it a “soft”, polarizable atom with significant inductive effects. All of the above allows for a multidimensional analysis of the substitution effects. The first dimension is formed by the choice of one fixed substituent –Br, placed in one or two sites along the carbon core. The second dimension is formed by the presence of one or two hydroxyl groups, with associated hydrogen bonding to carbonyl oxygen atoms in positions 1 and 4. Thus, a thorough picture of the molecular diversity in the considered molecules will be achieved.

Numerous 1,4-naphthoquinone derivatives have been investigated via diffractometry methods, as revealed in the Cambridge Crystallographic Data Center (CCDC) [56] database search. Bromination of naphthazarin was discussed as early as 1927 [57]. These structures (selected examples of CCDC deposition numbers: 720974, 976852, 1115404, 1137317, 1137406, 1164545, 1190358, 1526294, 1543933, 1859287) served as a reference of the possible substitution patterns, and we will discuss them in this light below. We underline, however, that our conceptual study has been devoted to the specific concept of bromine and hydroxy derivatization of 1,4-naphthoquinone (the parent 1,4-naphthoquinone was described structurally in 1965 [58]. This allowed us to examine the substituent effects without interference from other factors. The literature experimental data show that the 1,4-naphthoquinone derivatives have been drawing attention of scientists due to their molecular features. They have found applications of chemical nature, e.g., as ligands in coordination chemistry [59], and they are frequently found in nature, e.g., as elements of redox cycles, members of the vitamin K family [60], and plant ingredients such as lawsone [59]. They are interesting candidates for the use in medicinal chemistry, e.g., against cancer cells [61,62,63] and antifungal and antibacterial agents [64,65]. Materials chemistry uses this type of compound as ingredients of molecular magnets [66]. Diverse synthetic routes are still developed for novel derivatives of this class [67,68,69]. The potential for intermolecular interactions of diverse nature—from stacking to hydrogen bonding—makes these compounds interesting for co-crystallization studies [70].

Summarizing the introductory remarks, we underline that the questions for this study to answer are: (a) What is the impact of the –Br substituent on the given set of descriptors? (b) What is the role of the –O-H group (which cannot be treated as a classical substituent because it is engaged in intramolecular hydrogen bonding, introducing major changes to both the substituting entity as well as the core compound)? (c) What is the combined effect of the two substitution types in different spatial relations? These questions are crucial for the rational design of molecular systems with desired properties for the materials science, especially in light of the fact that the chosen molecules, possessing aromatic and antiaromatic rings with carbonyl functionalization, are non-trivial models for a broad spectrum of diverse chemical systems. The possibility of cooperative motions of the hydrogen bridges can introduce additional depth to the analysis, as the cooperation of hydrogen bonds is commonly found in real-life examples of many systems [71,72]. In order to answer these questions, Density Functional Theory (DFT) [73,74] and Car–Parrinello Molecular Dynamics (CPMD) [75] were employed. The methods allowed the examination of the influence of substitution on the molecular structure, as well as discrimination between the direct and indirect effects on the various molecular features.

## 2. Results and Discussion

### 2.1. Calculation and Analysis of Selected Physico-Chemical Parameters of the Studied 1,4-Naphthoquinone and Its Derivatives on the Basis of Static Density Functional Theory (DFT) Models

#### 2.1.1. Substituent Effect—Structure and Correlation Analyses

At the beginning of the structural analysis, as a support for the computational study, we have compared the literature structural data for the parent compounds of the studied groups **1**, **2** and **3**, with the results of the DFT calculations at the ωB97XD/6-311++G(2d,2p) level of theory. The relevant data for the heavy atom skeletons (with bonds to hydrogen atoms omitted) are gathered in Table 2, showing good agreement between the X-ray structures and the chosen theoretical level. In light of our planned property analysis, the most important feature of this agreement is adequate reproduction of the alternating pattern of bond lengths, indicative of the modulation of aromaticity. A detailed description of substituent effects also requires that the results for bromo-derivatives are validated. This is carried out in Table 3 for compounds **2b** and **2b(M)**, for which experimental structures are available. The comparison of X-ray and calculated structures shows that the C-Br bond is very well reproduced at the given level of theory, and the bond alteration is also correctly reproduced. The asymmetric part of the unit cell of the crystalline **2b** contains two independent molecules [62], and the differences in the bond lengths arising from the crystal packing show the degree of elasticity of the naphthoquinone skeleton. Moreover, this is also an important rationale for our choice of studying isolated molecules rather than crystals: substituent effects can be at least partially obscured by the environmental effects. For this reason, we also omit detailed discussion of solid-state phenomena such as halogen bonding, which could occur due to the presence of C-Br functions in the studied set of compounds.

The structural effect of substitution will be discussed on the basis of the aromaticity index HOMA [4], proton transfer energy barrier and the energy second minimum (the latter two in anticipation of the next section, where the intramolecular hydrogen bond metric parameters and the proton transfer energy profiles will be discussed in detail). All of the data were obtained as a result of the DFT simulations at the ωB97XD/6-311++G(2d,2p) level of theory, already mentioned above. First, let us consider the HOMA index as the overall indicator of the structural changes in the aromatic and antiaromatic fused rings. In the all studied cases, the changes of the HOMA index in the aromatic rings (substituted in groups **2** and **3** by the donor hydroxyl groups) are small. The details of the analysis are presented in the Supplementary Material as Tables S1–S3. As is shown in Table S1, the introduction of the –Br substituent did not significantly affect the benzene ring denoted as II in Figure S1. A slightly different situation was noticed for group **2** of the studied compounds. The presence of the –O-H group as the second substituent (see Table S2) decreased the aromaticity of the benzene ring to ca. 0.95. It is worth mentioning that as a consequence of the presence of the –O-H group, an intramolecular hydrogen bond was formed and one quasi-ring appeared in the structure. In group **3** (see Table S3), a larger decrease in aromaticity was observed of the benzene ring. The HOMA index is equal ca. 0.87 in the studied set of compounds. The presence of the –Br substituent as well as two hydroxyl groups affected the aromaticity. The introduction of two hydroxyl group resulted in the presence of two intramolecular hydrogen bonds and, associated with this, the formation of two quasi-rings.

**Figure 2:**
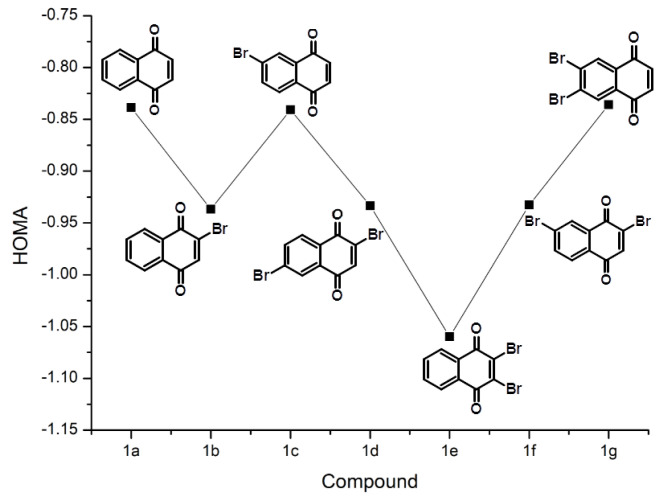
The HOMA aromaticity index evolution of the quinoid ring as a result of substitution by –Br in the compounds of group **1**.

**Figure 3:**
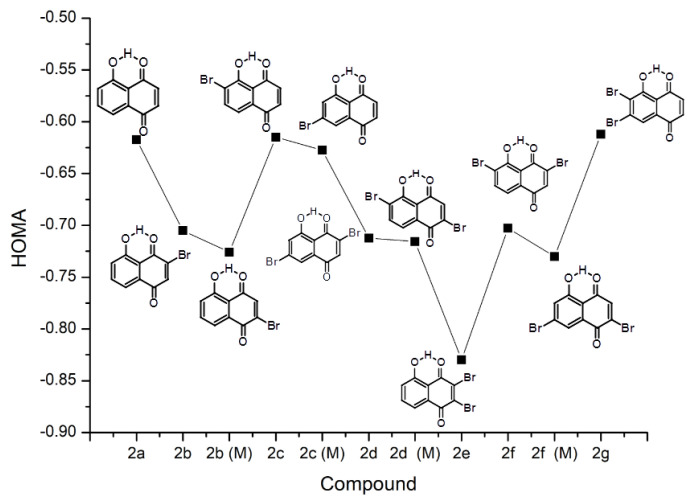
The HOMA aromaticity index evolution of the quinoid ring as a result of substitution by –Br and hydroxyl group in the compounds of group **2**.

**Figure 4:**
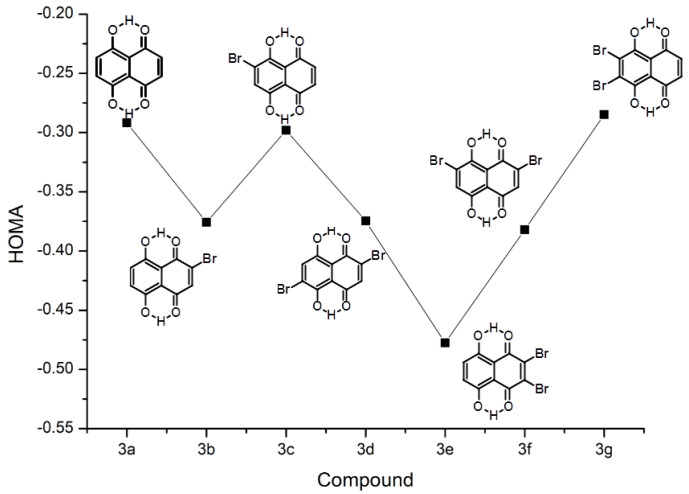
The HOMA aromaticity index evolution of the quinoid ring as a result of substitution by –Br and hydroxyl groups in the compounds of group **3**.

The quinoid (denoted as I in Figure S1) ring seems to be more affected by the presence of substituents, which is visible in the following Figures: Figure 2 for group **1**, Figure 3 for group **2**, and finally Figure 4 for group **3**. The most striking feature of these figures is that the relative changes in the HOMA index inside the group are very well conserved between the groups, even if the absolute values differ. The substitution patterns **a** (no substitution), **c** and **g** maintain basically the same HOMA level for the quinoid ring. These patterns cover substitution of the aromatic ring, and the conclusion must be drawn that the modifications of the aromatic moiety are not strongly transmitted to the quinoid ring. The Figure 3 shows that some transmissions take place because the HOMA values for mirror pairs, such as **2b** and **2b(M)** or **2c** and **2c(M)**, slightly differ. These differences, however, seem to originate in transmission through the O–H...O bridge. The deepest changes occur when the HOMA substituent is placed directly at the quinoid ring (patterns **b**, **d**, **e**, **f**). The HA index changes towards more localized (less antiaromatic) structures. The role of substituents depends on the type of the ring they are attached to—when the substitution patterns **e** and **g** are compared, it is visible that the substitution by two –Br atoms at the quinoid ring makes it more localized, while the same substitution in the aromatic ring slightly delocalizes the ring with carbonyl functions.

**Figure 5:**
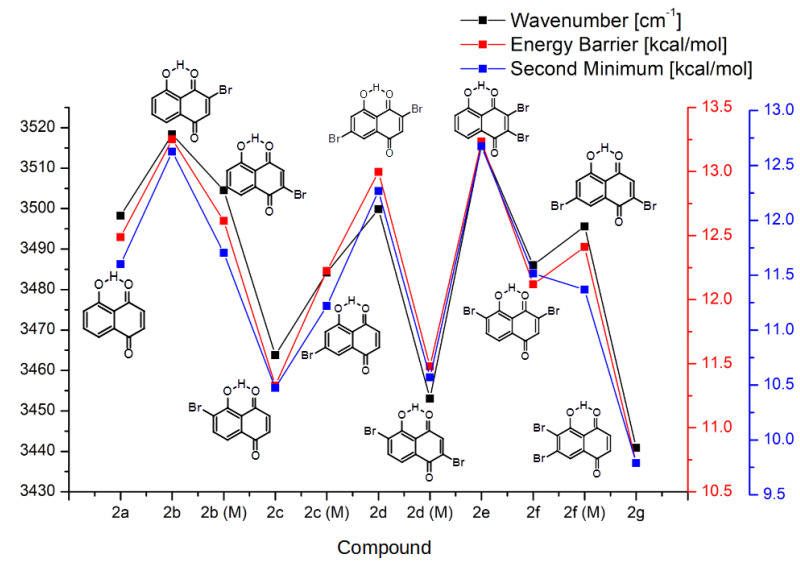
The $\nu_{OH}$ stretching wavenumber, energy barrier and the second minimum energy values in group **2** of the studied compounds.

**Figure 6:**
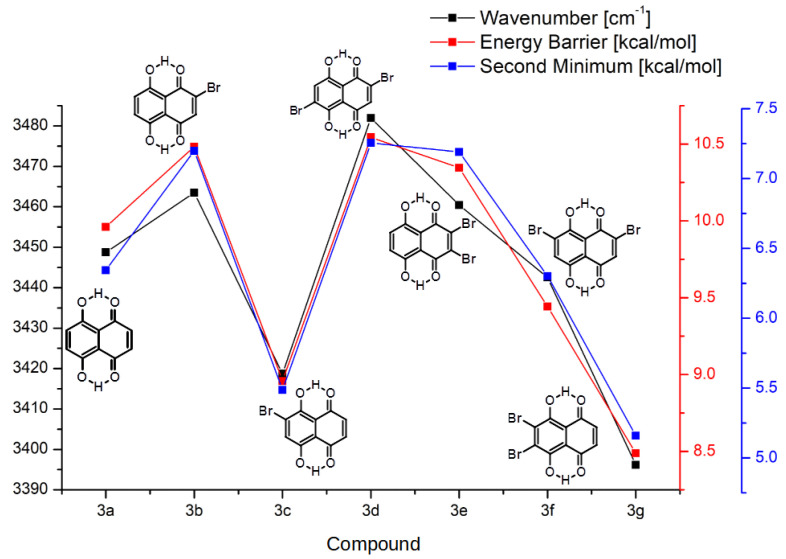
The $\nu_{OH}$ stretching wavenumber, energy barrier and the second minimum energy values in group **3** of the studied compounds.

Another manifestation of the substituent effect is visible in the properties of the hydrogen bridges; therefore, it can be analyzed only for groups **2** and **3**. It is again important to note that the overall shape of the property dependencies agrees well between groups **2** and **3**, as well as with the HOMA index discussed above. This shows that the fundamental mechanism of action of the bromine substituents is the same and affects diverse molecular properties in a similar manner. In particular, as shown in Figure 5 and Figure 6, a decrease in the aromaticity (i.e., increase in the atomic localization) of the quinoid ring is associated with the increase in the proton transfer barrier height and energy of the second minimum. The harmonic wavenumbers of the bridge OH stretching modes are also inversely correlated with the aromaticity of the quinoid ring. The comparison of the wavenumber ranges for the corresponding compounds of groups **2** and **3** shows that the presence of the second hydrogen bridge in group **3** lowers the band positions quite systematically by 50 cm${}^{-1}$. The details of the computed wavenumbers (corresponding to the harmonic approximation) for the OH stretching are presented in Tables S4 and S5.

#### 2.1.2. Intramolecular Hydrogen Bond and the Proton Reaction Path

The metric parameters of the intramolecular hydrogen bonds are presented in Tables S6 and S7, respectively. Let us start the discussion with group **2** of the studied compounds. As shown in Table S6, the O1...O3 interatomic distance ranges from 2.58 to 2.60 Å, while the hydrogen bond length ranges from 1.70 to 1.73 Å. The valence angle difference is equal ca. $2^{\circ }$ depending on the –Br substituent position. In the case of group **3**, two hydroxyl groups were introduced to the 1,4-naphthoquinone giving, as a result, the naphthazarin molecule. Therefore, the 1,4-naphthoquinone molecule is heavily structurally modified, because the presence of the two hydroxyl groups introduced two intramolecular hydrogen bonds, two quasi-rings and, moreover, it is one of many dihydroxynaphthoquinone structural isomers. In addition, to make our study more comprehensive, the –Br substituent (as the second one) was added and the inductive effect’s influence on the intramolecular hydrogen bonding was studied. In Table S7, the metric parameters of both hydrogen bonds of naphthazarin and its derivatives are presented. Let us consider the naphthazarin molecule as a reference structure to estimate the induction effect provided by the –Br substituent. As is presented in Table S7, the metric parameters of the hydrogen bridge are affected by the presence and position of the bromine. The changes could be considered small but noticeable. The intramolecular hydrogen bond is shortened when the –Br substituent is in its direct neighbourhood (see metric parameters of O...O and H...O obtained for compounds **3b**, **3c** and **3f**). An interesting situation was noticed for the **3d** molecule. The –Br substituents are located on the acceptor side and this hydrogen bridge is longer when compared to the second one (with the –Br substituent located on the proton-donor side). The diversity of the substitution patterns provides a large number of opportunities to observe effects of particular substituents on the proton transfer (PT) profile in the hydroxyl-containing groups **2** and **3**. The details of the reaction energetic outcomes are given in Tables S8 and S9, respectively. The PT profiles, grouped in Figure S2, exhibit several interesting features. First, dihydroxy series **3** generally displays lower PT barriers (by 2–3 kcal/mol) and an even lower position of the second minimum (by ca. 4–5 kcal/mol) with respect to series **2**. These facts indicate that the second hydrogen bridge, together with the second quasi-aromatic ring, act as molecular “buffers” alleviating the loss of aromaticity during the PT event. This effect goes even further—the O-H bond lengths at the barrier and at the second minimum are closer to each other in series **2** when compared with the corresponding group **3** compounds. This structural phenomenon can be interpreted in the following way: the twin hydrogen bridges in group **3** are coupled and the protons tend to be on the same side. When the PT event is enforced during the proton reaction path, this coupling must be overcome, and the energy increases from the first energy minimum to the energy barrier, which is more steep—so the barrier is reached faster with smaller O-H bond lengths.

In the PT reaction path of group **2** (Figure 5), it is apparent that the energies of the energy barrier and the second minimum in the proton transfer event are similar to the base, non-substituted compound **1a** when R1 and R4 are:

- Both occupied by hydrogens.
- Both occupied by bromine atoms.

The highest energies of the barrier and the second minimum belong to the compounds with bromine substituted in position R1 and hydrogen in the R4 position, namely **2b**, **2d** and **2e**. The lowest values of aforementioned parameters occur while the substitution is reversed—the hydrogen in R1 and bromine in R4 for the compounds **2c**, **2d(M)** and **2g**. The R2 and R3 bromine substitution seems to have very small effect on the PT. This behavior could mean that the bromine substitution has a very small effect mediated through the bonds in the ring, and the modulation of the PT transfer occurs mostly due to the inductive effect on either the donor or acceptor atoms (oxygen in the studied case) free electron pairs (or both, canceling each other’s effects). In group **3** (Figure 6), only the base compound **3a** and symmetrically substituted **3f** exhibit corresponding energies. Similarly to group **2**, the lowest energies occur in compounds **3c**, **3d** and **3g** (R4 substituted) and the highest energies were observed in compounds **3b** and **3e** (R1 substituted). This further backs the inductive effect of bromine substitution when close to the donor/acceptor side. It should be emphasized, however, that the PT reaction paths investigation were performed with only one of the hydrogen bridges. This gave us an overall picture of the energy of the proton reaction path in both studied groups of the compounds. However, in order to complete our discussion, Car–Parrinello Molecular Dynamics (CPMD) simulations were performed for group **3** and the proton dynamics in both hydrogen bridges was studied.

#### 2.1.3. Electronic Structure Analysis on the Basis of Hirshfeld Charges Distribution and Substituent Active Region (cSAR) Parameter

**Figure 7:**
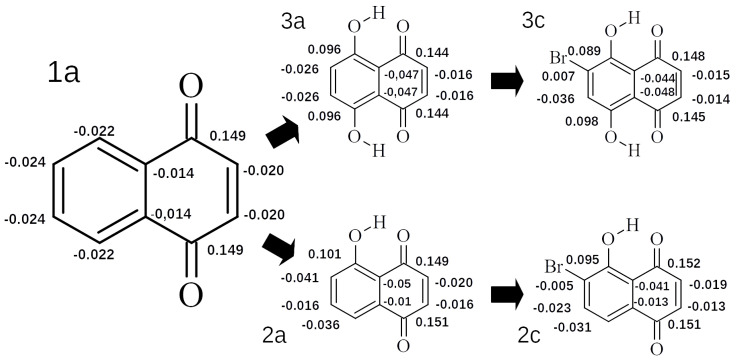
The Hirshfeld atomic charge distribution in 1,4-naphthoquinone and its exemplary derivatives. The values of carbon atoms are given.

To evaluate the influence of the –Br substitution on the electron density distribution in the fused rings, the Hirshfeld charge population analysis was performed. Figure 7 presents the example of the –Br influence on the atomic charge value in the aromatic and antiaromatic rings, for single, double and no hydrogen bridges. The main body of the data can be found in the Supplementary Information in Tables S10 and S11, respectively.

From this example it is clear that –Br mostly affects the carbon atom at the substitution site and, to a smaller extent, its closest neighbors. The effect on all other carbon atoms is negligible, especially in the case of two O-H groups. This could mean that the most pronounced effects on the hydrogen bridges is not mediated through the electron density redistribution in the rings, but by the inductive effect. This would imply that the proximal sides (R1 and R4 for O1–H1...O3 and R2 and R3 for O2–H2...O4) have, by far, the biggest influence on the bridge dynamics in a single mobile bridge scenario, which is consistent with the proton reaction path results.

The Hirshfeld atomic charges also serve to obtain the Substituent Active Region parameter (cSAR) [15], which is the sum of the atomic charges of a given substituent and the C(ipso) atom bearing this atom/group. The results are presented in Table S12. The use of a whole fragment including the C(ipso) atom allows for detecting the overall transfers of electron density over the cyclic skeleton and associated substituents. In the case of the quinoid ring, the negative values of cSAR for positions 1 and 4 are reported for all the investigated compounds, witnessing the electronegativity of the carbonyl oxygen atom (and its proton-accepting properties in groups **2** and **3**). Interestingly, all the remaining cSAR values are positive (with only a few exceptions—position 7 in group **2**). On the other hand, introduction of the –O-H groups at positions 5 and 8 makes the cSAR at these atoms only slightly modified with respect to the parent structures of group **1**. The impact of the –Br substituents is more pronounced. However, the correlations between the structure and the cSAR values are not easily discernible.

### 2.2. Time Evolution of Intramolecular Hydrogen Bonds Present in Selected 1,4-Naphthoquinone Derivatives on the Basis of Car–Parrinello Molecular Dynamics (CPMD)

The CPMD simulations of the compounds of group **3** were carried out in the gas phase to detect the substituent influence on the dynamical properties, especially those connected with the hydrogen bridge dynamics. The use of gas phase (isolated molecule) models allows for elimination of the environmental (crystal lattice) effects and better estimation of possibly subtle modifications introduced by the –Br atoms. The analysis of CPMD results covers time evolution of the bridge parameters (O-H...O distances) and vibrational signatures (atomic velocity power spectra).

**Figure 8:**
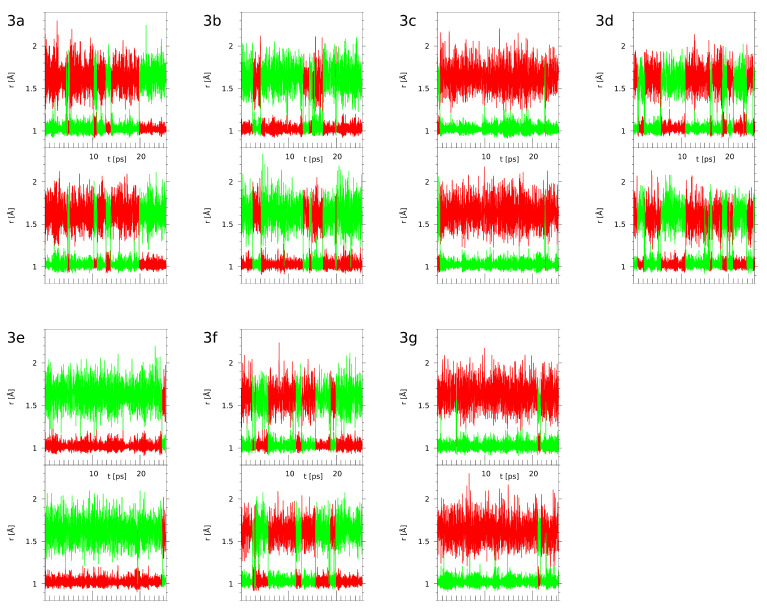
Time evolution of the O-H distances in the hydrogen bridges—CPMD simulation results for the compounds of group **3**. Red lines—donor-proton (O${}^{1}$-H${}^{1}$ or O${}^{2}$-H${}^{2}$) distances, green lines—acceptor-proton (H${}^{1}$-O${}^{3}$ or H${}^{2}$-O${}^{4}$) distances according to the starting structures drawn in Figure 1. For each compound, the upper part of its graph corresponds to the O${}^{1}$-H${}^{1}$-O${}^{3}$ bridge and the lower part to the O${}^{2}$-H${}^{2}$-O${}^{4}$ bridge.

The CPMD scheme allows reproduction of the interactions between the hydrogen bridges, which—as already mentioned above—extends the scope of molecular study beyond one-dimensional proton potential profiles. Indeed, the time evolution of the donor-proton and proton-acceptor distances, shown in Figure 8, at first glance reveals that the proton motions are heavily correlated between the bridges. Any PT event is recorded in both bridges at the same time. Our earlier study on naphthazarin [39] has shown that this process is not ideally synchronous, but the delay between the PT events in the two bridges is only a few OH stretching periods, less than 0.1 ps. The current study shows the same behavior of correlated, but not ideally synchronous, proton motions in the two bridges of a given molecule.

Valuable insight into the role of the –Br substituents is gained when we take a closer look at structurally similar molecules. In particular, compounds **3b** and **3c** differ so that in **3b** the –Br substituent is located close to the acceptor site, while in **3c**—close to the donor group. The same holds for **3e** (two –Br atoms on the acceptor side) and **3g** (the donor side is doubly substituted). The relation between **3d** and **3f** is such that both the donor and acceptor side are monosubstituted, but in **3d** the two substituents are positioned at different bridges, while in **3f** only one bridge is surrounded by the –Br atoms. Detailed discussion of the substituent effects will be based not only on the graphical representation of time evolution from Figure 8, but also on the proton possession data grouped in Table 4. The proton possession was evaluated on the simplest basis: the proton is, at a given moment, assigned to the closer of the two centers (donor or acceptor). Since the simulation times could be shorter than required for full ergodicity, the proton possession data will be discussed relative to the naphthazarin, compound **3a**, the parent structure of group **3**, devoid of the bromine substituents.

The bromine atom as a substituent promotes proton transfer (decreases pK${}_{a}$) not only in the carboxylic acids, but also in phenols. The pK${}_{a}$ of phenol is ca. 10, but pK${}_{a}$ of 2-bromophenol is 8.45. Figure 2, Figure 3, Figure 4, Figure 5 and Figure 6 and Figure S2 have already shown how the substituent position affects the host molecule, but incorporation of the dynamical and time-evolution aspects could reveal unexpected similarities. The data from the Figure 8 and Table 4 indicate that the –Br substituents act more strongly on the bridge proton dynamics when placed on the donor, not the acceptor, side. It is most visible in the pair **3b** (–Br at the acceptor side) and **3c** (donor side). Compound **3c** exhibits almost total proton transfer to the acceptor side (proton possession by the O${}^{1}$ and O${}^{2}$ donor atoms falls below 3%), while the protons in structure **3b** are located for ca. 80% of the simulation time on the donor atom side. Another similar pair of compounds is **3e** (double substitution at the acceptor side) and **3g** (the donor side). It takes two –Br substituents proximal to the acceptor atoms to keep the bridge protons on the donor side (almost 97% possession), while the **3g** compound keeps its bridge protons at the donor sites for only 3% of the time (pointing to the saturation of the substituent effect: the impact of two –Br atoms in **3g** is similar to one –Br atom in **3c**). The symmetry of these values, adding up to 100%, is not accidental: from the dynamical point of view, **3e** and **3g** correspond to the same compound, because the **3g** structure in a short time experiences a double PT event and converts into **3e**. These results lead to the question: what would happen when two –Br atoms would be placed in such positions as to achieve mutual cancelling out of the substituent effect? Structures **3d** and **3f** are built according to this notion. While the static calculations indicate that **3d** has significantly higher PT barrier than **3f** (see Figure 6, we have to remember that only one proton reaction path was studied), the CPMD simulation shows that these compounds share the property of having the bridge protons almost equally divided between the donor and acceptor sides. This fact requires further analysis: the **3d** structure contains one –Br substituent in proximal position to the donor group of one bridge, and another –Br atom in proximal position to the acceptor atom of the other bridge, while in **3f** the –Br atoms are in close contact with one bridge only—yet these two compounds possess the same type of proton bridge dynamics. This shows how strong the correlation between the two bridges is, and this will lead us to the notion of the secondary (cooperative) substituent effect, outlined in the next paragraph.

As already established from the DFT PT scans, a bromine atom’s influence on the single bridge acts in two possible ways: when substituted closest to the donor site (R4), it lowers the barrier for the process, while substitution closest to the acceptor site (R1) raises the barrier. This translates to raising and lowering the PT statistical probability, respectively. Addition of the second bridge dynamics changes the behavior drastically. As predicted, substitution proximal to the acceptor will promote the molecular forms, while being proximal to the donor will promote the PT form. However, the unique nature (mutual coupling) of the two intramolecular hydrogen bridges allows additional effects of substitution. While in the single bridge scans substitution on the distant sites R2 and/or R3 (with R1 and R4 occupied by hydrogen) is almost negligible for the O1–H1...O3 bridge, CPMD shows that the cooperation of the proton transfers in the bridges introduces major cross-input to the substituent effect. This is visible in the O2–H2...O4 dynamics—for this bridge, positions R1 and R4 are not the proximal sites; thus, they should not affect its dynamics in any major way. The CPMD results of compounds **3b** and **3c** show, however, that this is not the case. In **3b**, the molecular form of this bridge is preferred, while in **3c**, the PT form is prevalent. This is further supported by the most interesting case of the **3d** compound. With positions R1 and R3 occupied, the O1–H1...O2 bridge should prefer the molecular form, while the others should prefer the PT form. The cooperative nature results, however, in almost equal possession of the proton between the sites for both bridges. This compensation corresponds well with the cross-influence in **3b** and **3c**. We propose the mechanism of the secondary (or cooperative) substituent effect, mediated not by the typical resonance or inductive phenomena, but rather by the associated dynamics of the twin bridges.

**Figure 9:**
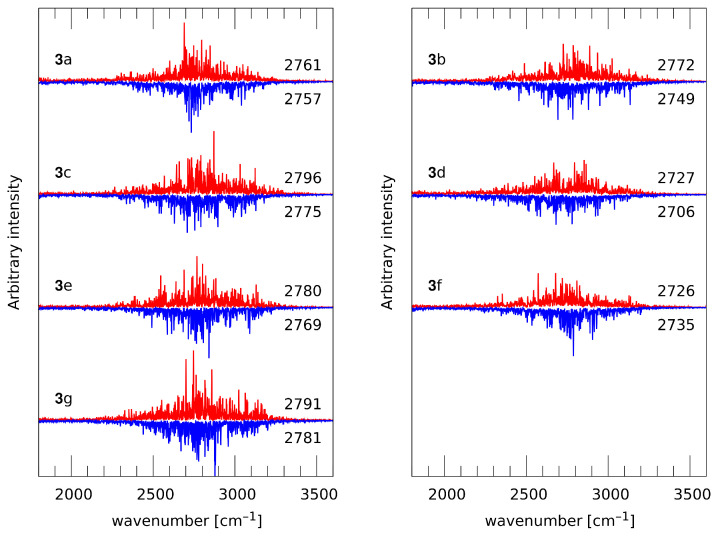
The bridge proton O-H stretching regions of the atomic velocity power spectra—CPMD simulation results for the compounds of group **3**. Red lines—H${}^{1}$ protons, blue lines—H${}^{2}$ protons. Barycenters (in cm${}^{-1}$) of each signature are given to the right of each graph.

Substituent effects influencing the bridge proton dynamics must have a noticeable impact on the vibrational signatures of the O${}^{1}$-H${}^{1}$ and O${}^{2}$-H${}^{2}$ groups. This is indeed the case, as evidenced in Figure 9. The numerical data provided in this figure are positions of the $\nu_{OH}$ barycenters and are significantly shifted towards smaller wavenumbers than the harmonic wavenumbers of Figure 6. This red shift stems from the dynamical nature of the atomic velocity power spectra, which—classical in terms of nuclear dynamics—are able to capture some effects of the underlying anharmonic potential energy surface. However, the registered changes agree very well with the harmonic data. Proceeding with the analysis of the CPMD vibrational features, at the beginning we will assess the convergence of the CPMD simulations, since the substituent effects could be subtle. The compounds with symmetrical locations of substituents, making the bridges equivalent, are **3a** (naphthazarin), **3e** and **3g**. The differences between the barycenters of the O${}^{1}$-H${}^{1}$ and O${}^{2}$-H${}^{2}$ stretching features in these compounds are, respectively, 4, 11 and 10 cm${}^{-1}$. The remaining compounds exhibit significantly larger splitting of bridge 1 vs. bridge 2 barycenters, exceeding 20 cm${}^{-1}$, which means that the substituent effects are present and significant. The only exception is compound **3f** (only 9 cm${}^{-1}$ split), where—on the basis of the distance time evolution and proton possession data—the presence of the secondary, cooperative substituent effect is evident, making the $\nu_{OH}$ of both bridges most affected (shifted towards lower wavenumbers). Interestingly, structure **3d**, with the $\nu_{OH}$ being the most red-shifted in the investigated series (bridge 1 by 35 cm${}^{-1}$ and bridge 2 by 55 cm${}^{-1}$, with respect to the parent structure **3a**), has virtually the same $\nu_{OH}$ for the O${}^{1}$-H${}^{1}$-O${}^{3}$ bridge as the structure **3f** (2727 vs. 2726 cm${}^{-1}$). The corresponding bridge 2 parameters for **3d** and **3f** are more diversified: 2706 vs. 2735 cm${}^{-1}$. This shows that the cooperative substituent effect is indeed less efficient than the effect of proximal –Br substitution.

## 3. Computational Methodology

### 3.1. Static Density Functional Theory (DFT) Calculations

The models of 1,4-naphthoquinone and its derivatives were constructed on the basis of experimental data available in the Cambridge Crystallographic Data Centre (CCDC) [56] (CCDC Database Identifier: NAPHQU01; Deposition Number 939729) [21]. For the crystallographic data visualization the Mercury 3.1 program was used [77]. 1,4-Naphthoquinone, denoted as the parent compound, was substituted by bromine and O-H group in diverse position and as a result 25 models were obtained (for details, see Figure 1 and Table 1). The models were built using the Molden 5.7 program [78]. The geometry optimization was performed using Density Functional Theory (DFT) [73,74] with ωB97XD functional [79] and valence-split triple-zeta Pople’s style basis set denoted as 6-311++G(2d,2p) [80,81]. Harmonic frequencies were computed and analyzed to confirm structure agreement with minimum on the Potential Energy Surface (PES). Next, the Harmonic Oscillator Model of Aromaticity (HOMA index) [20] was calculated for both rings. The HOMA index belongs to the class of geometric descriptors and it was obtained on the basis of the equation:

(1) $$HOMA=1-\frac{\alpha }{n}\sum_{i=1}^{n}{\left(d_{opt}-d_{i}\right)}^{2}$$

where *n* means the number of CC bonds taken into account, α = 257.7 is an empirical normalization constant chosen to give HOMA = 0 for non-aromatic system and HOMA = 1 for a system where all bonds are equal to $d_{opt}$ = 1.388 Å and $d_{i}$ are the bond lengths.

Subsequently, the Hirshfeld atomic charges [82] were computed and the Substituent Active Region (cSAR) parameter was obtained based on them [15]. It can be calculated according to the equation:

(2) $$cSAR\left(X\right)=q\left(X\right)+q\left(C_{ipso}\right)$$

by summing up atomic charges belonging to the substituent X and the ipso carbon atom to which the substituent is attached. Finally, for the optimized structures from groups **2** and **3** (see Figure 1), the proton reaction path was investigated in the hydrogen bridge denoted as O${}^{1}$—H${}^{1}$...O${}^{3}$. The scan with optimization method was applied for this purpose (the O-H increment was set to 0.05 Å, the valence angle was frozen and the rest of the structure was optimized). The quantum-chemical simulations were carried out with the Gaussian 16, Rev. C.01 suite of programs [83]. The obtained computational data was analyzed taking into account the metric parameters of the hydrogen bond, spectroscopic signatures and electronic structure.

### 3.2. Car–Parrinello Molecular Dynamics (CPMD) Simulations

Car–Parrinello molecular dynamics simulations for naphthazarin and its six mono- and di-bromosubstituted derivatives (group **3** of the studied compounds) were carried out in the gas phase. The choice of structures with two hydrogen bridges was dictated by our aim of detecting the influence of substitutions at the position of the bridged protons and the correlation of their movements. Initial structures were taken from the DFT ωB97XD/6-311++G(2d,2p) optimizations and were not optimized further before the CPMD run. The PBE functional [84] was used as the DFT exchange-correlation approximation, and the plane-wave basis set was terminated at the kinetic energy cutoff of 80 Ry. The Troullier–Martins norm-conserving pseudopotentials [85] and empirical Grimme D2 dispersion correction [86] were employed as further elements of the electronic structure setup. A monomeric molecule was placed in a cubic box with an 18 Å edge; this size was used for all molecules to retain the same conditions of CPMD runs. Interactions with periodic images were removed with the Hockney scheme [87] to attain the gas phase conditions. The temperature of 300 K was enforced by Nosé–Hoover thermostat chains [88,89] coupled to each atomic species (i.e., element) separately. The time step of 3 a.u. (0.0726 fs) was used to propagate the Car–Parrinello Lagrangian, and the data were collected every 20 steps. The spurious translational and rotational movement was also removed every 20 MD steps. The simulation lasted for 360,000 steps (26.1 ps), and the first 10,000 steps (0.7 ps) were taken as the equilibration (thermalization) time, and as such were excluded from the final analysis. The post-processing of the MD trajectories was carried out with the VMD 1.9.3 [90] and Gnuplot [91] programs as well as with the locally written scripts and utilities. The Car–Parrinello MD simulations were performed using the CPMD, ver. 4.3 suite of programs [92].

## 4. Conclusions

This case study concerns three groups of 1,4-naphthoquinone derivatives diversely substituted with the –Br atom and –O-H group (the latter arranged to form intramolecular hydrogen bridges with the carbonyl functions). The aromaticity of the two rings was investigated with the HOMA aromaticity index. The impact of the –Br substituents on the aromatic ring was found to be subtle in groups **1** and **2**; however, in the third group, a larger decrease in aromaticity was observed. The aromaticity index is equal ca. 0.88 in the group of the double-hydrogen bonded derivatives. The quinoid antiaromatic ring was affected rather strongly, with the exact effect dependent on the number and position of substituents. The intramolecular hydrogen bridge properties (the energy barrier height and the energy of the second minimum, harmonic wavenumbers of the $\nu_{OH}$ modes) were correlated, showing qualitatively the impact of the substitution. Strict correlations between these parameters were found, in particular, the fact that an increase in the barrier height is associated with the increase in the energy of the secondary minimum of the PT form, an increase in the $\nu_{OH}$ harmonic wavenumber, and a decrease in the quinoid ring HOMA index (increase in the orbital localization). Moreover, an interesting case of the two substituents acting either in opposition or in synergy in compounds of group **3** has shown that the overall effect is determined by the coupling between two hydrogen bridges. Finally, Car–Parrinello Molecular Dynamics simulations for the structures with two intramolecular hydrogen bridges have revealed important correlations between the bridge proton motions, further confirming the results of the static approach based on structure optimization. This fact, visible in the proton possession data and vibrational features, strongly suggests the presence of the secondary (cooperative) substituent effect, in which the impact of the substituents is transmitted through coupling between the affected groups. This results in the observed behavior of the proton dynamics, especially when the two –Br substituents counteract each other—the proton possession by the donor and the acceptor is then almost perfectly balanced. In the studied cases, the effects of the –Br substituents on the bridged proton dynamics are stronger when the substituents are placed on the donor, rather than the acceptor side. These results show the potential role of the type and placement of substituents in the smart design of building blocks for functional materials such as molecular switches and relays.

## Figures and Tables

**Table 1:** Arrangement of substituents in the studied compounds—see Figure 1.

Compounds	R${}_{1}$	R${}_{2}$	R${}_{3}$	R${}_{4}$
**1a**, **2a**, **3a**	–H	–H	–H	–H
**1b**, **2b**, **3b**	–Br	–H	–H	–H
**2b(M)**	–H	–Br	–H	–H
**1c**, **2c**, **3c**	–H	–H	–H	–Br
**2c(M)**	–H	–H	–Br	–H
**1d**, **2d**, **3d**	–Br	–H	–Br	–H
**2d(M)**	–H	–Br	–H	–Br
**1e**, **2e**, **3e**	–Br	–Br	–H	–H
**1f**, **2f**, **3f**	–Br	–H	–H	–Br
**2f(M)**	–H	–Br	–Br	–H
**1g**, **2g**, **3g**	–H	–H	–Br	–Br

**Table 2:** Selected experimental (X-ray) and calculated (ωB97XD/6-311++G(2d,2p) level) bond lengths (in Å) for the parent compounds **1a**, **2a**, **3a**—see Figure 1 for the atom numbering scheme and structural overview.

Compounds	1a, X-ray [21]	1a, DFT	2a, X-ray [76]	2a, DFT	3a, X-ray [22]	3a, DFT
C1=O	1.222	1.211	1.257	1.228	1.247	1.231
C4=O	1.223	1.211	1.205	1.211	1.240	1.231
C8-O	–	–	1.329	1.332	1.508	1.331
C5-O	–	–	–	–	1.341	1.331
C1-C2	1.474	1.484	1.437	1.479	1.478	1.477
C2-C3	1.332	1.331	1.350	1.331	1.343	1.332
C3-C4	1.473	1.484	1.478	1.484	1.478	1.447
C4-C10	1.486	1.491	1.500	1.492	1.476	1.459
C10-C5	1.393	1.389	1.339	1.378	1.402	1.391
C5-C6	1.384	1.385	1.413	1.396	1.417	1.412
C6-C7	1.388	1.390	1.389	1.376	1.384	1.364
C7-C8	1.382	1.385	1.416	1.400	1.430	1.412
C8-C9	1.394	1.389	1.388	1.404	1.309	1.391
C9-C10	1.404	1.397	1.380	1.408	1.433	1.421
C1-C9	1.490	1.491	1.477	1.463	1.461	1.459

**Table 3:** Selected experimental (X-ray) and calculated (ωB97XD/6-311++G(2d,2p) level) bond lengths (in Å) for the bromo-derivatives **2b** and **2b(M)**—see Figure 1 for the atom numbering scheme and structural overview.

Compounds	2b, X-ray [62]		2b, DFT	2b(M), X-ray [67]	2b(M), DFT
	mol.1	mol.2			
C1=O	1.224	1.231	1.222	1.240	1.228
C4=O	1.207	1.205	1.211	1.218	1.205
C8-O	1.348	1.357	1.330	1.346	1.331
C1-C2	1.487	1.483	1.495	1.471	1.476
C2-Br	1.869	1.887	1.880	–	–
C3-Br	–	–	–	1.876	1.878
C2-C3	1.334	1.318	1.330	1.341	1.331
C3-C4	1.474	1.478	1.481	1.492	1.500
C4-C10	1.492	1.491	1.489	1.488	1.493
C10-C5	1.379	1.398	1.377	1.386	1.378
C5-C6	1.400	1.384	1.396	1.397	1.396
C6-C7	1.388	1.368	1.375	1.383	1.376
C7-C8	1.382	1.379	1.400	1.402	1.400
C8-C9	1.411	1.404	1.406	1.405	1.404
C9-C10	1.417	1.408	1.407	1.411	1.406
C1-C9	1.451	1.454	1.464	1.462	1.461

**Table 4:** Percentages of proton possession by the donor atoms in both hydrogen bridges of each investigated compound of group **3**—results of the CPMD simulation.

Compound	O${}^{1}$–H${}^{1}$	O${}^{2}$–H${}^{2}$
**3a**	66.9	67.7
**3b**	79.3	77.5
**3c**	2.6	2.7
**3d**	44.3	47.7
**3e**	96.8	96.7
**3f**	53.2	51.7
**3g**	3.1	3.1

## Data Availability

The data presented in this study are available in the article itself and in the Supplementary Materials.

## Supplementary Materials

The supplementary Tables S1–S12 and Figures S1–S2 are available online at https://www.mdpi.com/article/10.3390/ijms221910357/s1.

## Abbreviations

The following abbreviations are used in this manuscript:

SE	Substituent effect
3D	Three-dimensional
DFT	Density Functional Theory
HOMA	Harmonic Oscillator Model of Aromaticity
CCDC	Cambridge Crystallographic Data Center
PES	Potential Energy Surface
cSAR	Substituent Active Region parameter
PT	Proton transfer
CPMD	Car–Parrinello Molecular Dynamics
MD	Molecular dynamics
