# Supplementary material for: How Substitution Combines with Non-Covalent Interactions to Modulate 1,4-Naphthoquinone and Its Derivatives Molecular Features—Multifactor Studies
Source: Int J Mol Sci. 2021 Sep 26;22(19):10357. doi: 10.3390/ijms221910357 (PMC8508802; doi:10.3390/ijms221910357)
Supplement: Supplementary file 1 [file ijms-22-10357-s001.zip › ijms-1387037-supplementary.pdf]

SUPPLEMENTARY INFORMATION

How Substitution Combines with Non-covalent Interactions to Modulate 1,4-naphthoquinone and its Derivatives Molecular Features – Multifactor Studies

Michał Pocheć[1†], Karol Kułacz[1†], Jarosław Panek[1], Aneta Jezierska[1]*

[1] Faculty of Chemistry, University of Wrocław, ul. F. Joliot-Curie 14, 50-383 Wrocław, Poland

[†]These authors contributed equally to this work

Correspondence: aneta.jezierska@chem.uni.wroc.pl; Tel.: +48 71 3757 224; fax: +48 71 3282 348

Table of content

I. **Figure S1.** The structures of the investigated compounds with the atoms and rings numbering scheme prepared for the current study. The dotted line indicates the presence of intramolecular hydrogen bond.

II. **Table S1.** HOMA aromaticity index for the group 1. The geometry optimization was performed at the ωB97XD/6-311++G(2d,2p) level of theory. For the atoms and rings numbering scheme see Figure S1.

III. **Table S2.** HOMA aromaticity index for the group 2. The geometry optimization was performed at the ωB97XD/6-311++G(2d,2p) level of theory. For the atoms and rings numbering scheme see Figure S1.

IV. **Table S3.** HOMA aromaticity index for the group 3. The geometry optimization was performed at the ωB97XD/6-311++G(2d,2p) level of theory. For the atoms and rings numbering scheme see Figure S1.

I. **Table S4.** Wavenumbers and intensities for the OH stretching maxima for the group 2 obtained at the ωB97XD/6-311++G(2d,2p) level of theory.

II. **Table S5.** Wavenumbers and intensities for the OH stretching maxima for the group 3 obtained at the ωB97XD/6-311++G(2d,2p) level of theory.

III. **Table S6.** Intramolecular hydrogen bond metric parameters for the group 2 obtained at the ωB97XD/6-311++G(2d,2p) level of theory.

IV. **Table S7.** Intramolecular hydrogen bonds metric parameters for the group 3 obtained at the ωB97XD/6-311++G(2d,2p) level of theory.

V. **Figure S2.** Proton potential functions of the O1-H1…O3 hydrogen bridge for the studied compounds from groups 2 and 3, calculated at the ωB97XD/6-311++G(2d,2p) level of theory.

VI. **Table S8.** Energy barrier and second energy minimum obtained at the ωB97XD/6-311++G(2d,2p) level of theory for the group 2 as a result of scan with optimization.

VII. **Table S9.** Energy barrier and second energy minimum obtained at the ωB97XD/6-311++G(2d,2p) level of theory for the group 3 as a result of scan with optimization.

VIII. **Table S10.** The values of partial atomic charges of carbon atoms, computed according to the Hirshfeld scheme at the ωB97XD/6-311++G(2d,2p) level of theory. For atoms numbering scheme see Figure S1.

IX. **Table S11.** The values of partial atomic charges of oxygen and hydrogen atoms, computed according to the Hirshfeld scheme at the ωB97XD/6-311++G(2d,2p) level of theory. For atoms numbering scheme see Figure S1.

X. **Table S12.** cSAR values of carbon atoms of the fused rings. The values of partial atomic charges of carbon atoms were computed according to the Hirshfeld scheme at the ωB97XD/6-311++G(2d,2p) level of theory. For atoms numbering scheme see Figure S1.

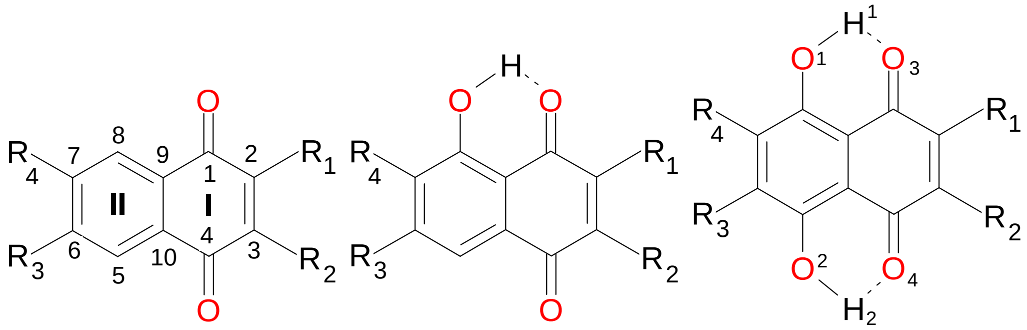

Figure S1. The structures of the investigated compounds with the atoms and rings numbering scheme prepared for the current study. The dotted line indicates the presence of intramolecular hydrogen bond.

Table S1. HOMA aromaticity index for the group 1. The geometry optimization was performed at the ωB97XD/6-311++G(2d,2p) level of theory. For the atoms and rings numbering scheme see Figure S1.

Compound	Ring I	Ring II
**1a**	-0.839	0.995
**1b**	-0.937	0.996
**1c**	-0.841	0.995
**1d**	-0.933	0.996
**1e**	-1.060	0.998
**1f**	-0.933	0.996
**1g**	-0.836	0.995

Table S2. HOMA aromaticity index for the group 2. The geometry optimization was performed at the ωB97XD/6-311++G(2d,2p) level of theory. For the atoms and rings numbering scheme see Figure S1.

Compound	Ring I	Ring II
**2a**	-0.617	0.953
**2b**	-0.705	0.948
**2b (M)**	-0.726	0.956
**2c**	-0.615	0.952
**2c (M)**	-0.627	0.957
**2d**	-0.712	0.952
**2d (M)**	-0.716	0.955
**2e**	-0.830	0.955
**2f**	-0.703	0.946
**2f (M)**	-0.730	0.960
**2g**	-0.612	0.951

M indicates the mirrored substitution pattern (along the long axis)

Table S3. HOMA aromaticity index for the group 3. The geometry optimization was performed at the ωB97XD/6-311++G(2d,2p) level of theory. For the atoms and rings numbering scheme see Figure S1.

Compound	Ring I	Ring II
**3a**	-0.292	0.876
**3b**	-0.376	0.872
**3c**	-0.298	0.879
**3d**	-0.375	0.875
**3e**	-0.478	0.875
**3f**	-0.382	0.873
**3g**	-0.285	0.874

Table S4. Wavenumbers and intensities for the OH stretching maxima for the group 2 obtained at the ωB97XD/6-311++G(2d,2p) level of theory.

Compound	OH stretching	
	**ν [cm^{-1}]**	**I [km/mol]**
**2a**	3498	209
**2b**	3518	202
**2b (M)**	3504	221
**2c**	3463	261
**2c (M)**	3484	234
**2d**	3499	229
**2d (M)**	3453	282
**2e**	3515	216
**2f**	3486	253
**2f (M)**	3495	247
**2g**	3440	282

M indicates the mirrored substitution pattern (along the long axis)

Table S5. Wavenumbers and intensities for the OH stretching maxima for the group 3 obtained at the ωB97XD/6-311++G(2d,2p) level of theory.

**Compound**	**OH stretching**	
	**ν [cm^{-1}]**	**I [km/mol]**
**3a**	3448	204
**3b**	3463	146
**3c**	3418	158
**3d**	3405	164
**3e**	3460	202
**3f**	3442	179
**3g**	3396	259

Table S6. An intramolecular hydrogen bond metric parameters for the group 2 obtained at the ωB97XD/6-311++G(2d,2p) level of theory.

Compound	O1…O3	O1-H1	H1…O3	<O1H1O3
**2a**	2.5986	0.9788	1.7282	146.10
**2b**	2.5906	0.9777	1.7255	145.41
**2b(M)**	2.6018	0.9786	1.7326	145.96
**2c**	2.5830	0.9807	1.7061	146.78
**2c(M)**	2.5999	0.9794	1.7291	146.07
**2d**	2.5921	0.9783	1.7267	145.38
**2d(M)**	2.5848	0.9806	1.7094	146.56
**2e**	2.5876	0.9779	1.7230	145.30
**2f**	2.5770	0.9793	1.7060	146.03
**2f(M)**	2.6029	0.9792	1.7339	145.85
**2g**	2.5802	0.9820	1.6994	147.19

Table S7. Intramolecular hydrogen bonds metric parameters for the group 3 obtained at the ωB97XD/6-311++G(2d,2p) level of theory.

Compound	O1…O3	O1-H1	H1…O3	<O1H1O3	O2…O4	O2-H2	H2…O4	<O2H2O4
**3a**	2.5903	0.9805	1.7188	145.99	2.5891	0.9805	1.7175	146.00
**3b**	2.5808	0.9795	1.7141	145.37	2.5889	0.9807	1.7183	145.83
**3c**	2.5726	0.9823	1.6947	146.65	2.5901	0.9806	1.7192	145.88
**3d**	2.5817	0.9796	1.7157	145.26	2.5697	0.9829	1.6911	146.68
**3e**	2.5738	0.9800	1.7069	145.30	2.5756	0.9801	1.7089	145.27
**3f**	2.5633	0.9813	1.6894	146.13	2.5906	0.9808	1.7204	145.74
**3g**	2.5653	0.9835	1.6824	147.25	2.5658	0.9835	1.6830	147.25

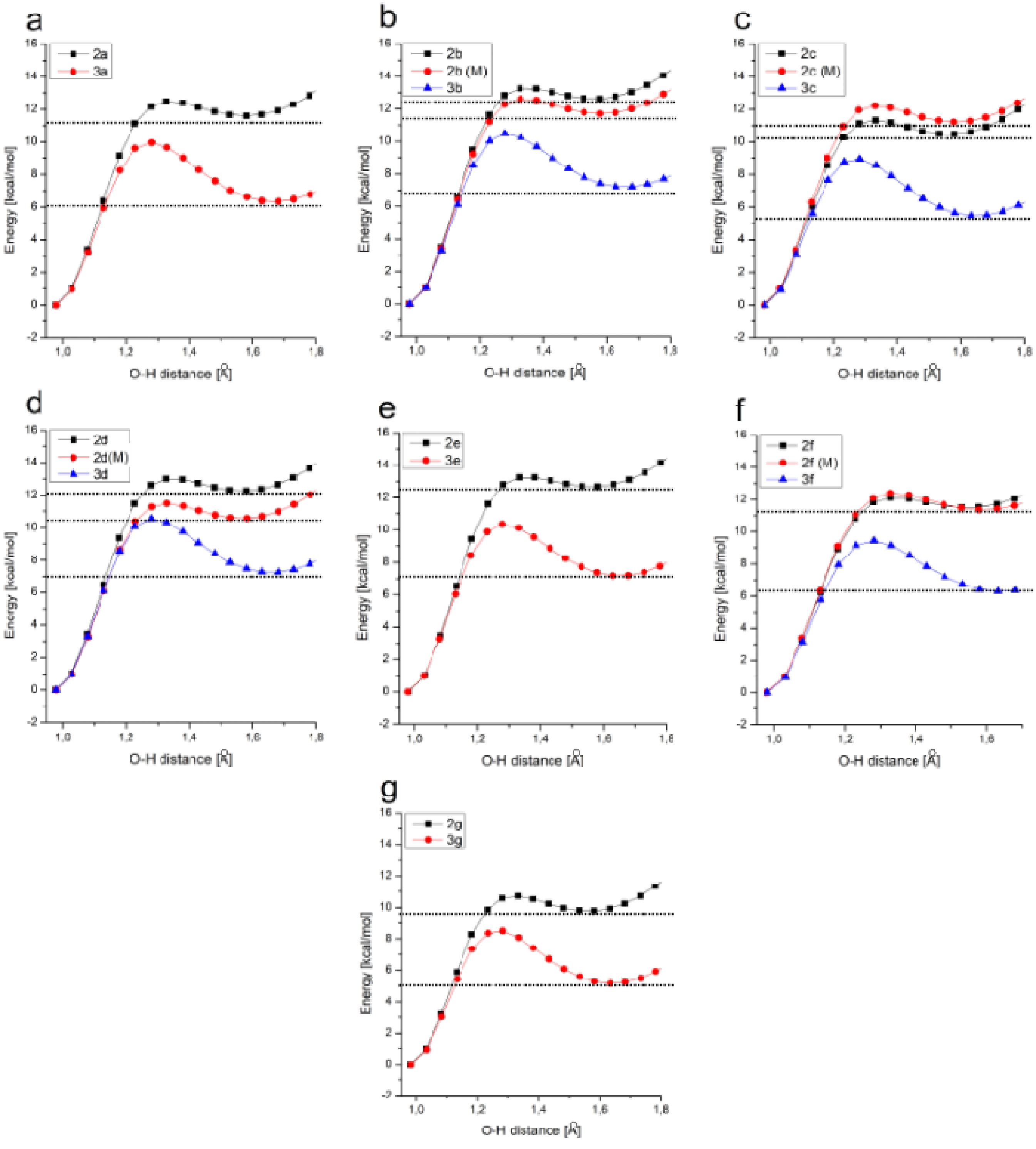

Figure S2. Proton potential functions of the O1-H1…O3 hydrogen bridge for the studied compounds from groups 2 and 3, calculated at the ωB97XD/6-311++G(2d,2p) level of theory.

Table S8. Energy barrier and second energy minimum obtained at the ωB97XD/6-311++G(2d,2p) level of theory for the group 2 as a result of scan with optimization.

Compound	O-H distance [Å]	Energy barrier [kcal/mol]	O-H distance [Å]	Second energy minimum [kcal/mol]	$dE_{(max-min)}$ [kcal/mol]
**2a**	1.3288	12.488	1.5788	11.600	0.888
**2b**	1.3277	13.251	1.5777	12.625	0.626
**2b (M)**	1.3286	12.616	1.5786	11.705	0.911
**2c**	1.3307	11.330	1.5807	10.475	0.855
**2c(M)**	1.3294	12.225	1.5794	11.220	1.005
**2d**	1.3283	12.997	1.5783	12.267	0.730
**2d (M)**	1.3306	11.479	1.5806	10.569	0.910
**2e**	1.3323	13.235	1.5823	12.674	0.561
**2f**	1.3293	12.119	1.5293	11.5150	0.604
**2f (M)**	1.3292	12.412	1.5792	11.3693	1.043
**2g**	1.3319	10.724	1.5819	9.7880	0.936

M indicates the mirrored substitution pattern (along the long axis)

$dE_{(max-min)} = E_{max} - E_{second\ minimum}$

Table S9. Energy barrier and second energy minimum obtained at the ωB97XD/6-311++G(2d,2p) level of theory for the group 3 as a result of scan with optimization.

**Compound**	**O-H distance [Å]**	**Energy barrier [kcal/mol]**	**O-H distance [Å]**	**Second energy minimum [kcal/mol]**	**$dE_{(max-min)}$ [kcal/mol]**
**3a**	1.2805	9.961	1.6805	6.344	3.618
**3b**	1.2795	10.484	1.6795	7.199	3.285
**3c**	1.2823	8.958	1.6323	5.486	3.472
**3d**	1.2796	10.544	1.6796	7.255	3.289
**3e**	1.2800	10.347	1.6300	7.190	3.157
**3f**	1.2813	9.442	1.6313	6.303	3.140
**3g**	1.2835	8.489	1.6335	5.159	3.329

$dE_{(max-min)} = E_{max} - E_{second\ minimum}$

Table S10. The values of partial atomic charges of carbon atoms, computed according to the Hirshfeld scheme at the ωB97XD/6-311++G(2d,2p) level of theory. For atoms numbering scheme see Figure S1.

C	1	2	3	4	5	6	7	8	9	10
**1a**	0.149	-0.020	-0.020	0.149	-0.022	-0.024	-0.024	-0.022	-0.014	-0.014
**1b**	0.148	0.009	-0.032	0.150	-0.020	-0.021	-0.022	-0.019	-0.013	-0.014
**1c**	0.150	-0.018	-0.019	0.152	-0.028	0.016	-0.029	-0.015	-0.014	-0.009
**1d**	0.149	0.011	-0.031	0.152	-0.026	0.018	-0.028	-0.013	-0.014	-0.008
**1e**	0.147	-0.007	-0.007	0.147	-0.018	-0.020	-0.020	-0.018	-0.013	-0.013
**1f**	0.150	-0.031	0.010	0.151	-0.026	0.017	-0.027	-0.013	-0.014	-0.008
**1g**	0.152	-0.017	-0.017	0.152	-0.024	0.007	0.007	-0.024	-0.011	-0.011
**C**	**1**	**2**	**3**	**4**	**5**	**6**	**7**	**8**	**9**	**10**
**2a**	0.149	-0.020	-0.016	0.151	-0.036	-0.016	-0.041	0.101	-0.045	-0.013
**2b**	0.147	0.009	-0.029	0.151	-0.033	-0.013	-0.040	0.104	-0.044	-0.012
**2b (M)**	0.149	-0.033	0.013	0.149	-0.033	-0.015	-0.039	0.103	-0.045	-0.012
**2c**	0.152	-0.019	-0.013	0.151	-0.031	-0.023	-0.005	0.095	-0.041	-0.013
**2c (M)**	0.150	-0.018	-0.014	0.154	-0.043	0.022	-0.049	0.105	-0.045	-0.007
**2d**	0.148	0.010	-0.028	0.154	-0.041	0.023	-0.048	0.108	-0.045	-0.007
**2d (M)**	0.152	-0.032	0.014	0.150	-0.029	-0.022	-0.003	0.096	-0.041	-0.013
**2e**	0.146	-0.007	-0.004	0.148	-0.032	-0.013	-0.038	0.105	-0.044	-0.012
**2f**	0.150	0.009	-0.027	0.151	-0.029	-0.021	-0.004	0.097	-0.041	-0.013
**2f (M)**	0.150	-0.031	0.013	0.152	-0.040	0.022	0.047	0.107	-0.045	-0.007
**2g**	0.152	-0.017	-0.013	0.154	-0.040	0.012	-0.016	0.098	-0.044	-0.010
**C**	**1**	**2**	**3**	**4**	**5**	**6**	**7**	**8**	**9**	**10**
**3a**	0.144	-0.016	-0.016	0.144	0.096	-0.026	-0.026	0.096	-0.047	-0.047
**3b**	0.142	0.012	-0.029	0.144	0.097	-0.024	-0.025	0.099	-0.047	-0.048
**3c**	0.148	-0.015	-0.014	0.145	0.098	-0.036	0.007	0.089	-0.044	-0.048
**3d**	0.142	0.014	-0.029	0.147	0.091	0.008	-0.036	0.100	-0.048	-0.044
**3e**	0.140	-0.004	-0.004	0.140	0.100	-0.023	-0.023	0.100	-0.048	-0.048
**3f**	0.145	0.012	-0.028	0.148	0.099	-0.034	0.007	0.092	-0.043	-0.049
**3g**	0.148	-0.014	-0.014	0.148	0.090	-0.006	-0.006	0.090	-0.047	-0.047

M indicates the mirrored substitution pattern (along the long axis)

Table S11. The values of partial atomic charges of oxygen and hydrogen atoms, computed according to the Hirshfeld scheme at the ωB97XD/6-311++G(2d,2p) level of theory. For atoms numbering scheme see Figure S1.

Oxygen atom	1	2	3	4	Hydrogen atom	1	2
1a	---	---	-0.238	-0.238	1a	---	---
1b	---	---	-0.222	-0.234	1b	---	---
1c	---	---	-0.235	-0.232	1c	---	---
1d	---	---	-0.220	-0.229	1d	---	---
1e	---	---	-0.221	-0.221	1e	---	---
1f	---	---	-0.232	-0.217	1f	---	---
1g	---	---	-0.231	-0.231	1g	---	---
**Oxygen atom**	**1**	**2**	**3**	**4**	**Hydrogen atom**	**1**	**2**
2a	-0.180	---	-0.221	-0.235	2a	0.129	---
2b	-0.177	---	-0.177	-0.208	2b	0.131	---
2b (M)	-0.178	---	-0.218	-0.220	2b (M)	0.130	---
2c	-0.173	---	-0.214	-0.232	2c	0.131	---
2c (M)	-0.175	---	-0.218	-0.228	2c (M)	0.131	---
2d	-0.172	---	-0.206	-0.226	2d	0.133	---
2d (M)	-0.171	---	-0.212	-0.218	2d (M)	0.132	---
2e	-0.176	---	-0.207	-0.219	2e	0.131	---
2f	-0.170	---	-0.202	-0.230	2f	0.133	---
2f (M)	-0.173	---	-0.216	-0.214	2f (M)	0.132	---
2g	-0.170	---	-0.213	-0.228	2g	0.131	---

Table S11 (Continuation). The values of partial atomic charges of oxygen and hydrogen atoms, computed according to the Hirshfeld scheme at the ωB97XD/6-311++G(2d,2p) level of theory. For atoms numbering scheme see Figure S1.

Oxygen atom	1	2	3	4	Hydrogen atom	1	2
**3a**	-0.180	-0.180	-0.226	-0.226	**3a**	0.129	0.128
**3b**	-0.176	-0.177	-0.213	-0.224	**3b**	0.131	0.130
**3c**	-0.173	-0.177	-0.218	-0.223	**3c**	0.130	0.130
**3d**	-0.173	-0.172	-0.211	-0.217	**3d**	0.132	0.131
**3e**	-0.175	-0.175	-0.212	-0.213	**3e**	0.131	0.131
**3f**	-0.170	-0.174	-0.206	-0.222	**3f**	0.132	0.131
**3g**	-0.172	-0.172	-0.217	-0.217	**3g**	0.130	0.130

Table S12. cSAR values of carbon atoms of the fused rings. The values of partial atomic charges of carbon atoms were computed according to the Hirshfeld scheme at the ωB97XD/6-311++G(2d,2p) level of theory. For atoms numbering scheme see Figure S1.

	cSAR(X)							
**X=**	**1**	**2**	**3**	**4**	**5**	**6**	**7**	**8**
**1a**	-0.089	0.042	0.042	-0.089	0.031	0.029	0.029	0.031
**1b**	-0.074	0.018	0.033	-0.084	0.035	0.033	0.032	0.035
**1c**	-0.085	0.046	0.045	-0.080	0.028	0.000	0.027	0.042
**1d**	-0.072	0.024	0.035	-0.077	0.030	0.007	0.029	0.045
**1e**	-0.074	0.015	0.015	-0.074	0.037	0.035	0.035	0.037
**1f**	-0.082	0.036	0.023	-0.066	0.030	0.006	0.030	0.045
**1g**	-0.079	0.048	0.048	-0.079	0.033	0.009	0.009	0.033
**X=**	**1**	**2**	**3**	**4**	**5**	**6**	**7**	**8**
**2a**	-0.071	0.044	0.049	-0.084	0.015	0.039	0.016	0.049
**2b**	-0.030	0.021	0.038	-0.057	0.019	0.043	0.019	0.058
**2b (M)**	-0.069	0.033	0.027	-0.070	0.019	0.042	0.020	0.054
**2c**	-0.062	0.046	0.053	-0.081	0.024	0.034	-0.012	-0.061
**2c (M)**	-0.068	0.048	0.052	-0.075	0.011	0.013	0.011	0.061
**2d**	-0.058	0.028	0.040	-0.073	0.014	0.019	0.014	0.068
**2d (M)**	-0.060	0.035	0.034	-0.068	0.027	0.036	-0.006	0.057
**2e**	-0.061	0.017	0.023	-0.071	0.022	0.045	0.022	0.060
**2f**	-0.052	0.026	0.041	-0.079	0.027	0.038	-0.007	0.060
**2f (M)**	-0.066	0.036	0.033	-0.062	0.014	0.018	0.108	0.065
**2g**	-0.060	0.049	0.055	-0.074	0.014	0.018	-0.007	0.059
**X=**	**1**	**2**	**3**	**4**	**5**	**6**	**7**	**8**
**3a**	-0.081	0.049	0.049	-0.081	0.044	0.036	0.036	0.044
**3b**	-0.070	0.051	0.052	-0.079	0.050	0.040	0.038	0.054
**3c**	-0.070	0.051	0.052	0.368	0.051	0.027	0.012	0.046
**3d**	-0.069	0.034	0.038	-0.069	0.050	0.019	0.029	0.060
**3e**	0.136	0.136	0.023	-0.072	0.056	0.041	0.041	0.056
**3f**	-0.061	0.032	0.040	-0.077	0.056	0.031	0.017	0.055
**3g**	-0.069	0.054	0.054	-0.069	0.048	0.013	0.013	0.048

X – carbon atom involved in the substitution
M indicates the mirrored substitution pattern (along the long axis)
